# The distribution of organic carbon fractions in a typical loess-paleosol profile and its paleoenvironmental significance

**DOI:** 10.7717/peerj.4611

**Published:** 2018-04-13

**Authors:** Qingqing Zhang, Jinghua Huang, Feinan Hu, Na Huo, Yingni Shang, Wenqian Chang, Shiwei Zhao

**Affiliations:** 1 State Key Laboratory of Soil Erosion and Dryland Farming on the Loess Plateau, Northwest A&F University, Yangling, Shaanxi, China; 2 State Key Laboratory of Soil Erosion and Dryland Farming on the Loess Plateau, Institute of Soil and Water Conservation, Chinese Academy of Sciences and Ministry of Water Resources, Yangling, Shaanxi, China; 3 University of Chinese Academy of Sciences, Beijing, China

**Keywords:** The Chunhua loess-paleosol sequence, Grain size, Paleoclimate, MOC/TOC, CaCO_3_

## Abstract

**Background:**

The loess-paleosol sequence on the Loess Plateau has been considered an important paleoclimatic archive to study global climatic and environmental changes in the Quaternary. So far, little attention has been paid to the characteristics of soil organic carbon fractions in loess-paleosol sequences, which may provide valuable information for exploring the evolution of climate and environment in the Quaternary on the Loess Plateau.

**Methods:**

In order to explore the significance of mineral-associated organic carbon to total organic carbon (MOC/TOC) ratios in the loess-paleosol sequence for reconstructing paleoenvironmental and paleoclimatic evolution in the Quaternary on the Loess Plateau, we selected a typical loess-paleosol profile in Chunhua county, Xianyang city, Shaanxi province, as the research object. The content of total organic carbon (TOC) and MOC/TOC ratio in each loess and paleosol layers of the Chunhua loess-paleosol profile were analyzed, together with the paleoclimatic proxies, such as soil grain size, CaCO_3_ content and their correlations with organic carbon parameters.

**Results:**

The main results were as follows: (1) the total content of soil organic carbon and MOC/TOC ratios were generally higher in paleosol layers than in the underlying loess layers of the Chunhua loess-paleosol profile. Compared to total organic carbon content, MOC/TOC ratios changed more obviously in soil layers below a paleosol layer S8; (2) soil clay content and median grain size (Md (*ϕ*)) were higher in paleosol than in the underlying loess, while CaCO_3_ content showed an opposite tendency. In the Chunhua profile, the distribution characteristics of the three paleoclimatic proxies showed good indications of paleoclimate changes in the Quaternary; (3) in the Chunhua loess-paleosol profile, MOC/TOC ratios were positively correlated with clay content and median grain size (*ϕ*), while negatively correlated with CaCO_3_ content, and the correlations were more significant in soil layers below S8.

**Discussion:**

Our results indicated that MOC/TOC ratios in the Chunhua loess-paleosol profile correlated with the cold dry-warm wet paleoclimatic cycle in the Quaternary. The high MOC/TOC ratios in the loess-paleosol profile might reflect warm and humid climate, while lower ratios indicated relatively cold and dry climate. That is because when the climate changed from warm-humid to cold-dry, the vegetation coverage and pedogenesis intensity decreased, which increased soil CaCO_3_ content and decreased soil clay content and Md (*ϕ*), leading to decreased MOC/TOC ratios. Compared to TOC, MOC/TOC ratios had greater significance in indicating paleoenvironmental evolution in the Quaternary on the Loess Plateau. Therefore, investigating MOC/TOC ratios in loess-paleosol profile can offer new evidence to reconstructing paleoenvironmental changes, and also provide a basis for predicting responses of soil organic carbon pools to vegetation and climate changes in the future.

## Introduction

As the best accessible paleoclimatic archives in terrestrial environments, the loess-paleosol stratigraphic sequences have been proven to preserve continuous paleoenvironment and paleoclimate records during the Quaternary, which are not only helpful for the reconstruction of regional and global paleoclimate evolution, but also provide vital references for the assessment of environmental and climatic changes in the future (An, 2000; Fitzsimmons, 2012; Fischer, 2012; Lehmkuhl et al., 2016). Over the past two decades, a series of researches have been carried out to study the characteristics of climate proxies in Chinese loess-paleosol sequences, such as soil grain size, CaCO_3_ content, magnetic susceptibility, organic carbon isotope, clay minerals and so on, which provided a wealth of information for the reconstruction of paleoenvironment and paleoclimate changes in the Quaternary (Verosub et al., 1994; Gallet, Jahn & Torii, 1996; Ding et al., 2002; Kohfeld & Harrison, 2003; Kaakinen, Sonninen & Lunkka, 2006; Torrent et al., 2007; Jeong, Hillier & Kemp, 2011; Liu et al., 2011; Krauß et al., 2016; Schulte & Lehmkuhl, 2017). Previous results have showed that the changes in soil grain size on a Chinese loess-paleosol profile could reflect the winter monsoon intensity and the distance between source areas and sedimentary areas (Chen et al., 1997; Nugteren & Vandenberghe, 2004; Sun et al., 2006; Prins et al., 2007; Guan et al., 2016; Zeng et al., 2017), and the distribution of CaCO_3_ could indicate the level of precipitation controlled by East Asian monsoon and reflect the weathering intensity in the loess-paleosol sequence (Chen et al., 1997; Zhao, Gu & Du, 2008; Babeesh et al., 2017). Therefore, soil grain size and CaCO_3_ content of loess-paleosol sequences have been widely used as the paleoclimatic proxies in the Quaternary studies. As an important component of soil, soil organic carbon (SOC) is fundamental in improving the physical, chemical, and biological functions of soil, and also in predicting the response of terrestrial carbon cycle to climate change (Kirschbaum, 2000). Previous studies have documented that the formation and transformation of organic carbon in soil are closely related to environmental factors, especially climate conditions (temperature and precipitation), which determine vegetation condition and soil microbial activity, and exert significant effects on the amount and fractions of soil organic carbon. So far, there have been studies investigating the distribution of organic carbon content in Chinese loess-paleosol profiles and its relationship with paleoclimatic changes. However, less attention has been paid to the characteristics of soil organic carbon fraction (e.g., mineral-associated organic carbon) in loess-paleosol sequences, which may provide valuable information for exploring the evolution of climate and environment on the Loess Plateau in the Quaternary.

It is widely known that the pool of soil organic carbon consists of different fractions, which greatly differ in stabilization mechanisms and turnover times. Among these fractions, particulate organic carbon (POC) is easily decomposed and extremely sensitive to environmental changes (Schmidt et al., 2011), which therefore occupy a relatively small proportion of total organic carbon (TOC) in soil. In contrast, mineral-associated organic carbon (MOC), which is stabilized by fine soil particles, accounts for large proportion of total SOC because of its high stability and long turnover time (Balesdent et al., 1998; Trumbore, 2000; Gregorich et al., 2006; Kahle, Kleber & Jahn, 2015). Therefore, MOC plays a critical role in SOC persistence. So far, the composition of organic carbon fractions in modern soil and its influencing factors have been extensively studied. It has been documented that the impact of climate variation on vegetation had significant effects on soil organic carbon content and fraction (Dobarco & Miegroet, 2014; Köchy et al., 2015; Cai et al., 2016; Wang et al., 2017; Puissant et al., 2017). In the absence of human disturbance, climate is a major constraint to the types and productivity of vegetation, which can lead to differences in the quantity, composition and decomposition rate of organic matter input coming from plants to soil, and further affect soil microbial biomass and activity (Wiesmeier et al., 2014; Köchy et al., 2015; Qin et al., 2016; Qin et al., 2017), inducing different changes in soil organic carbon fraction composition, especially the content and proportion of MOC (Triberti et al., 2008; Dobarco & Miegroet, 2014; Shang et al., 2015; Gabarrón-Galeote, Trigalet & Wesemael, 2015; Gao et al., 2016; Puissant et al., 2017). Cai et al. (2016) found that climate was the important regulating factor of MOC/TOC ratios. Puissant et al. (2017) also indicated that the content of MOC was significantly affected by the changes in temperature and precipitation condition due to the climate-induced variations in above-ground and below-ground plants biomass and soil microbial activity. Additionally, Yang, Niu & Wen (2012) have studied the relationship of POC in soil with vegetation characteristics and environmental factors at different altitudes on Helan Mountain in Ningxia province of China, and found that the POC/TOC ratios positively correlated with vegetation coverage, aboveground biomass and mean annual precipitation, while being negatively correlated with mean annual temperature. Therefore, it can be inferred indirectly from the results that the proportion of soil mineral-associated organic carbon in total organic carbon (MOC/TOC ratios) positively correlated with mean annual temperature, while being negatively correlated with mean annual precipitation, showing an opposite response tendency with POC/TOC to climatic changes. Similar results were also reported by Qi et al. (2016), which found that temperature warming significantly decreased the content of POC and its proportion in TOC, implying the increases in MOC/TOC in soil with the increasing temperature. These studies provided evidences that not only soil organic carbon content, but also the characteristics of soil organic carbon fractions, such as MOC/TOC ratio, were closely related to climatic conditions, which therefore could be considered as a potential proxy for climatic and environment conditions. Compared to modern soil, the proportion of MOC should be higher in loess-paleosol sequences because POC was continuously decomposed during the long-term burial. Therefore, studying the distribution of MOC/TOC in Chinese loess-paleosol sequence and its paleoenvironmental significance may better reflect the responses of SOC stock and dynamics to paleoclimate changes in the Quaternary, and is of great importance for reconstructing paleoenvironmental and paleoclimate evolution on the Loess Plateau.

In this study, the Chunhua loess-paleosol profile, which was an entirely and continuously developed profile, was selected as the research object. It is located in Chunhua county, Shaanxi province, China. We hypothesized that compared to the total organic carbon, the distribution of MOC/TOC in the loess-paleosol profile could better reflect paleoclimate and paleoenvironmental changes in the Quaternary. We investigated the distribution characteristics of soil organic carbon fractions (POC and MOC) in the Chunhua loess-paleosol profile, analyzed their correlations with two paleoclimatic proxies (soil grain size and CaCO_3_ content), and combined our results with previous studies about the changes of soil organic carbon fractions under modern climate and vegetation variations, to explore the significance of soil organic carbon fraction characteristics for the reconstruction of paleoenvironmental and paleoclimatic evolution in the Quaternary on the Loess Plateau.

## Materials and Methods

### Study site

**Figure 1:**
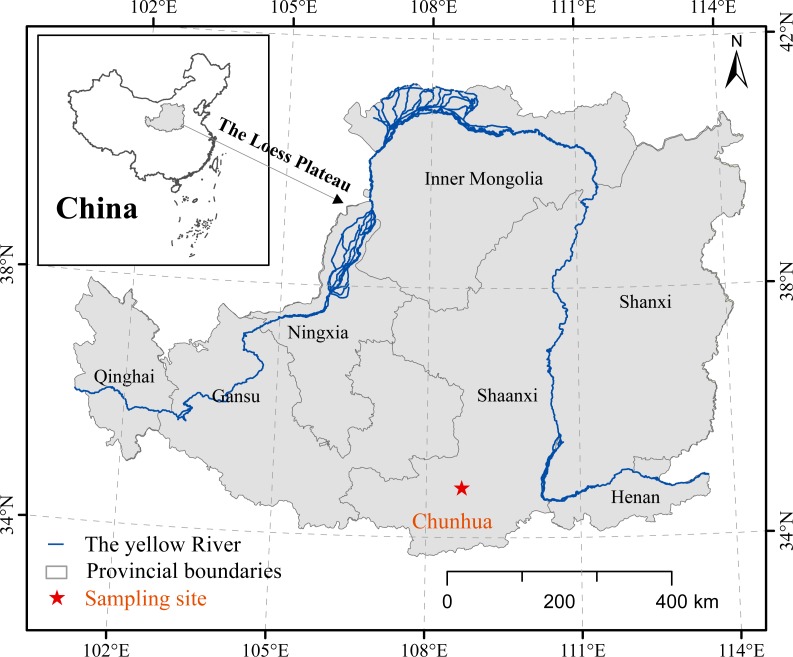
Location of the Chunhua loess-paleosol profile.

**Figure 2:**
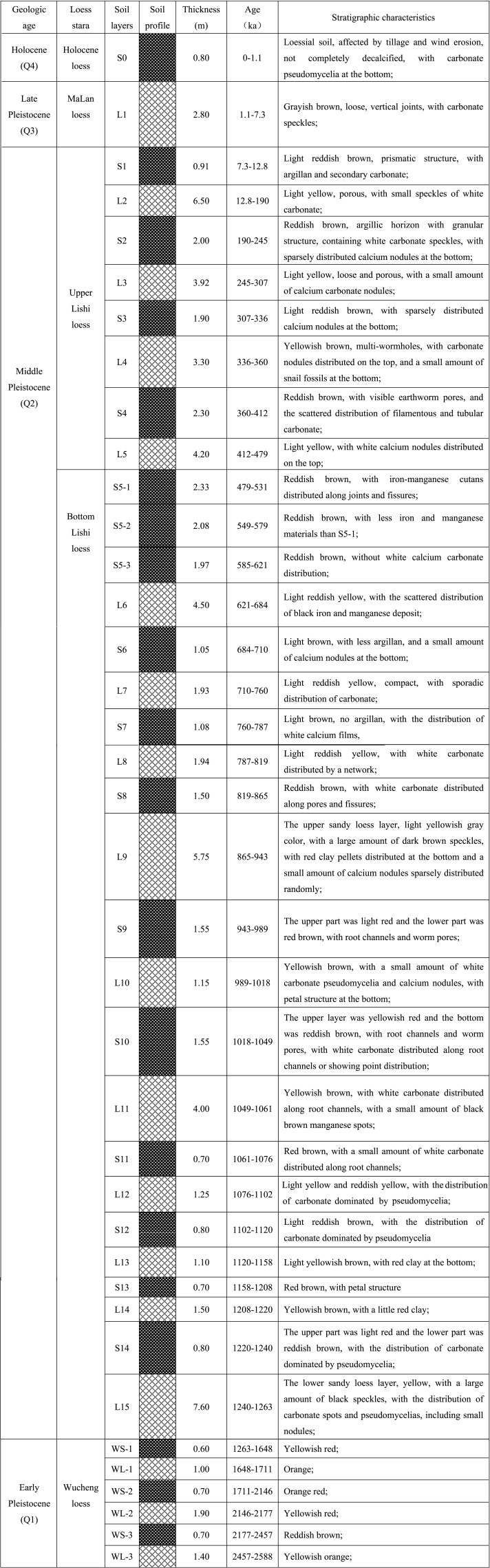
Histogram of Chunhua loess-paleosol profile with detailed description of basic characteristics of loess and paleosol layers.

The Chunhua loess-paleosol profile (34°48′07″N, 108°41′28″E) is located in the east of Chunhua county, which is in the northern part of Xianyang City, Shaanxi province, China (Fig. 1). This area is mainly affected by the East Asian monsoon, which belongs to the warm temperate semi-humid climate zone, with mean annual temperature of 10.4 °C and mean annual precipitation of 610 mm. The rainfall is mainly concentrated between July to September of each year. The total thickness of the Chunhua loess-paleosol profile is about 82 m, which consists of 18 loess layers and 18 paleosol layers (Fig. 2), belonging to a stable and continuous loess-paleosol sequence.

### Sample collection

From each loess and paleosol layer of the Chunhua profile, the loess or paleosol samples were collected. According to the layer thickness, three samples were uniformly collected from the top to bottom of each loess and paleosol layer, except S0 layer and S5 layer. At the sampling site, two land use managements, apple orchard and natural grassland, existed on the top layer (S0) of the Chunhua profile, which may exert different effects on the properties of the loessial soil in S0 layer. Therefore, in order to make a comparison, we collected samples of S0 layer under two land use managements: apple orchard (S0-1) and grassland (S0-2). Based on soil appearance characteristics, the composite paleosol layer S5 was divided into three layers: upper layer (S5-1), middle layer (S5-2) and under layer (S5-3), and soil samples were taken from each layer, respectively. Therefore, 117 samples were collected in total from the Chunhua loess-paleosol profile. The basic characteristics (age, thickness, and stratigraphic characteristics) of all loess and paleosol layers of the Chunhua profile were described in Fig. 2. Before laboratory analysis, all air-dried samples were sieved through a 2 mm mesh to remove coarse roots and stones, and the fine roots were picked out by the tweezers. Before the analysis of CaCO_3_ content and total organic carbon content, all paleosol and loess samples which have been sieved through a 2 mm mesh were further sieved through a 0.25 mm mesh in order to ensure the accuracy.

### Soil physicochemical analysis

Soil pH was determined based on water: soil ratio of 2.5:1 according to the standard method. The total CaCO_3_ content in paleosol and loess was analyzed by the use of the gas volume method (Dreimanis, 1962; Zhao et al., 2016). The concentration of hydrochloric acid (HCl) used in the gas volume method was 4 mol/L.

Soil grain size was analyzed by the sieving-sedimentation method (ISO, 1998). 10 g of paleosol or loess sample was weighed, and then washed by 0.2 mol/L hydrochloric acid and 10% hydrogen peroxide to remove carbonate and organic matter, respectively. Subsequently, soil pH was regulated to about 7.3 by using 0.5 mol/L sodium hydroxide. Finally, through ultrasonic dispersion, the 0.05–0.02 mm, 0.02–0.002 mm, and <0.002 mm particles were separately extracted according to the Stokes Law.

Soil total organic carbon (TOC) was measured using the improved potassium dichromate- external heating method (Nelson et al., 1996). 0.5000 g of paleosol or loess sample was weighed and placed in a hard glass tube, with the addition of 5 ml 0.8 mol/L potassium dichromate and 5 ml sulfuric acid solution. Then the mixture in the tube was heated in an oil bath at 180 °C for 5 min. After oxidative digestion, the remaining potassium dichromate was titrated with 0.2 mol/L ferrous ammonium sulfate, and according to the consumed amount of potassium dichromate, the content of total organic carbon was calculated.

Particulate organic carbon (POC) was determined according to the procedure as follows (Garten et al., 1999): 10.0 g of paleosol or loess sample was placed in a 100 ml centrifuge tube and dispersed in 50 ml of sodium hexametaphosphate solution (5 g/L). After 3 min of manual shaking, the soil suspension was shaken for 18 h by a horizontal shaker at a speed of 90 rpm, and then passed through 53 µm sieves. The sieves were washed repeatedly by distilled water, and all the soil left on the sieves was collected and weighed after oven-drying at 60 °C. The particulate organic carbon content in the oven-dried sample was determined using the improved potassium dichromate—external heating method. Mineral-associated organic carbon content (MOC) was the difference between soil total organic carbon content and particulate organic carbon content: *MOC* = *TOC* − *POC*.

### Statistical analysis

One-way ANOVA followed by the Duncan test was carried out to test the significant difference in soil properties among different loess and paleosol layers of the Chunhua loess-paleosol profile. Pearson correlation analysis was applied to test the relationships between soil properties across different layers of the Chunhua profile. In order to facilitate the analysis of the results, the median diameters (Md) of soil particles were log-transformed into *ϕ* values according to the Uddeh-Wentworth grade scale (Selly, 1985) prior to statistical analysis, and the conversion formula is: *ϕ* =  −Log_2_d (d—particle diameter, mm). Statistical significance was taken as *P* < 0.05 if not specially noted. All statistical analyses were performed using SAS 9.0 (SAS Institute Inc., Cary, NC, USA).

## Results

### Total organic carbon content

**Figure 3:**
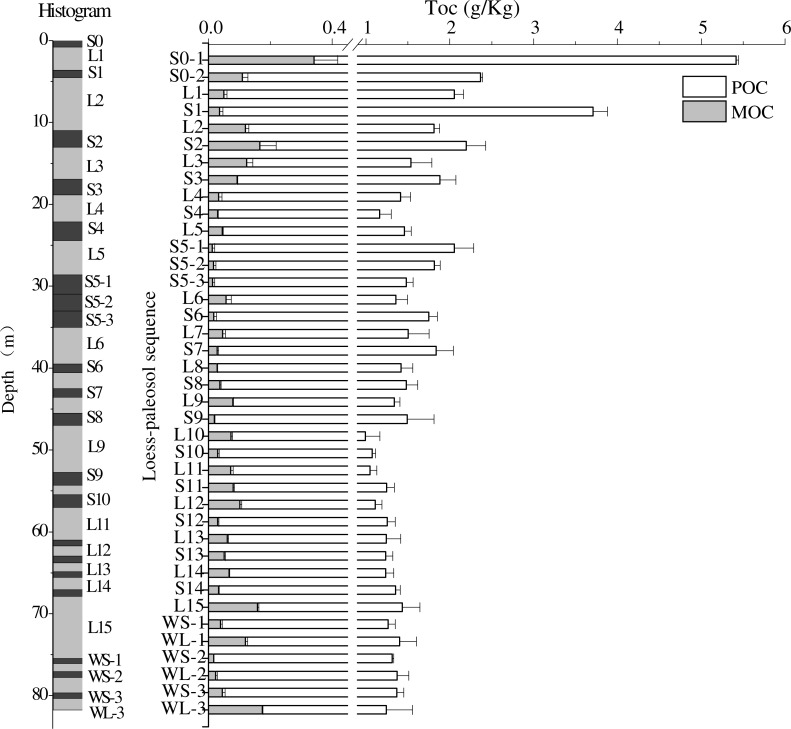
The contents of mineral-associated organic carbon and particulate organic carbon in loess and paleosol layers of Chunhua profile. Error bars represent the standard errors (SE).

The average content of soil total organic carbon in the Chunhua loess-paleosol profile was 0.16%, with the highest content in paleosol layer S0-1 and the lowest content in loess layer L10. In all paleosol layers of the Chunhua profile, total organic carbon content ranged from 0.11% to 0.54%, with the average value of 0.18%, while it was within the range of 0.10% to 0.21% in loess layers, with the average value of 0.14%. The results of variance analysis showed that total organic carbon content displayed significant differences between different soil layers of the Chunhua profile (*P* < 0.001), and were significantly higher in paleosol layers S0-1 and S1 than in other soil layers (Fig. 3). The difference in organic carbon content between different layers decreased in soil layers below paleosol layer S8, but still reached the significant level (*P* < 0.01). Generally, total organic carbon contents decreased with the increase of soil depth, while they were higher in paleosol layers than in the underlying loess layers (Fig. 3). In the soil layers below S8, the total organic carbon content tended to be more stable (Fig. 3).

### Mineral-associated organic carbon content and its proportion in total orgainc carbon

**Figure 4:**
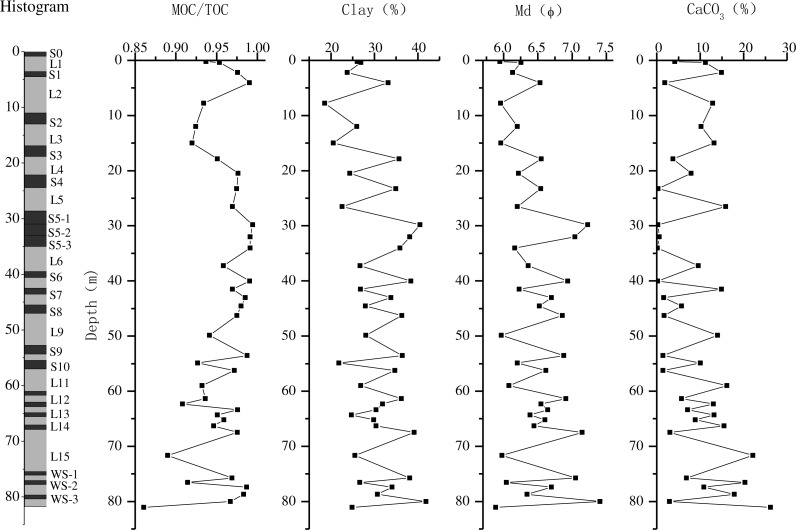
The distribution of the proportion of mineral-associated organic carbon in total organic carbon (MOC/TOC), clay content, median grain size (*φ*) and CaCO_3_ content in Chunhua loess-paleosol profile.

The average content of MOC in the Chunhua loess-paleosol profile was 0.16%. In the paleosol layers, MOC content was in the range of 0.10%–0.51%, with the average value of 0.18%, while it ranged from 0.08% to 0.20% in loess layers, with the average value of 0.13%. Similar to total organic carbon content, mineral-associated organic carbon contents in the paleosol layers were higher than those in the underlying loess layers (Fig. 4). The average MOC/TOC ratio was 0.971 in paleosol layers, with the highest value close to 1 (0.995), while it was 0.941 in loess layers (Fig. 4). Similar to TOC and MOC, MOC/TOC ratios were higher in paleosol layers than in the underlying loess layers, but in the soil layers below S8, the differences in MOC/TOC among different layers were more obvious (Fig. 4).

### Grain size distribution

**Figure 5:**
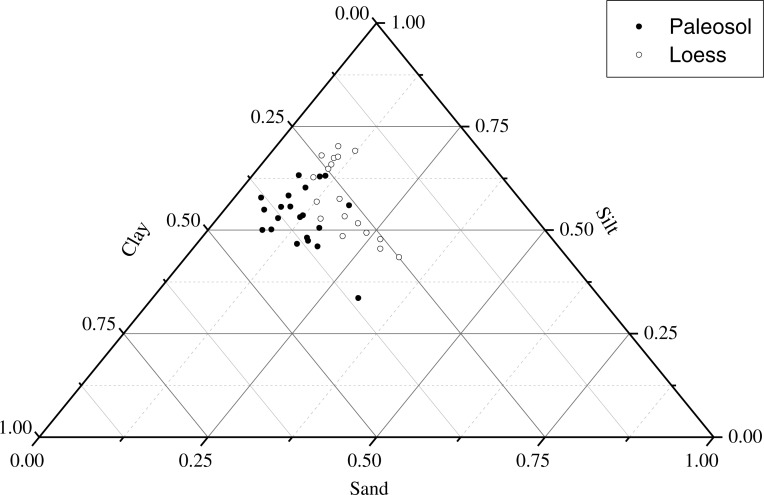
The distribution characteristics of soil grain size in paleosol layers (filled symbols) and loess layers (open symbols) of Chunhua loess-paleosol profile.

The grain size distribution in the Chunhua loess-paleosol profile is shown in Figs. 4 and 5. The particle composition of the Chunhua profile was as follows: silt was the main component, accounting for 43%–71% of soil, followed by clay, occupying 18%–42%, and sand content was the lowest (Fig. 5). The clay content in paleosol layers was in the range of 25%–42%, while it ranged from 18% to 32% in loess layers, and the clay content in each paleosol layer was higher than that in the underlying loess (Fig. 4). In the Chunhua loess-paleosol profile, the clay content generally increased with the increase of soil depth (Fig. 4). The median diameter Md (*ϕ* value) changed significantly among different soil layers. In general, median diameter Md (*ϕ*) in paleosol layers was higher than that in the underlying loess layer (Fig. 4), which showed a similar tendency across the Chunhua profile with clay content.

### CaCO_3_ content

The results showed that the average content of CaCO_3_ in all layers of the Chunhua profile was 8.93%. In paleosol layers, CaCO_3_ content was in the range of 0.15%–11.31%, while in the loess layers, it ranged from 5.71% to 26.18%. CaCO_3_ content differed significantly between different soil layers, and was obviously lower in paleosol layers than in the underlying loess layers (Fig. 4).

### Correlation between MOC/TOC ratio and paleoclimatic proxies

In general, MOC/TOC ratios showed significantly positive correlations with clay content and median diameter Md (*ϕ*) across all the layers of the Chunhua profile, and the correlation coefficients were 0.54 and 0.59 (Table 1), respectively, while being negatively correlated with the content of CaCO_3_, with the correlation coefficient of −0.71 (Table 1). In soil layers formed in different geologic periods, the correlations of MOC/TOC with clay content, Md (*ϕ*), and CaCO_3_ content varied. In the soil layers below paleosol layer S8, the correlations of MOC/TOC with paleoclimatic proxies, clay content, Md (*ϕ*) and CaCO_3_ content, were more significant, with the correlation coefficients reaching 0.63, 0.68 and −0.75, respectively (Table 2).

**Table 1:** Pearson correlation coefficients (*r*) between the proportion of mineral-associated organic carbon in total organic carbon and paleoclimate indicative indexes in Chunhua loess-paleosol profile.

	MOC/TOC	Clay	Md (*ϕ*)	CaCO_3_
MOC/TOC	1	0.54^**^	0.58^***^	−0.71^***^
Clay	0.54^**^	1	0.88^***^	−0.70^***^
Md (*ϕ*)	0.58^***^	0.88^***^	1	−0.68^***^
CaCO_3_	−0.71^***^	−0.70^***^	−0.68^***^	1

**Notes.**

** *P* < 0.01

*** *P* < 0.001.

**Table 2:** Pearson correlation coefficients (*r*) between the proportion of mineral-associated organic carbon in total organic carbon and paleoclimate indicative indexes in strata between the paleosol layer S8 and the loess layer WL-3 in Chunhua profile.

	MOC/TOC	Clay	Md (*ϕ*)	CaCO_3_
MOC/TOC	1	0.63^**^	0.68^**^	−0.75^**^
Clay	0.63^**^	1	0.92^***^	−0.75^**^
Md (*ϕ*)	0.68^**^	0.92^***^	1	−0.84^***^
CaCO_3_	−0.75^**^	−0.75^**^	−0.84^***^	1

**Notes.**

** *P* < 0.01.

*** *P* < 0.001.

## Discussion

### Distribution characteristics of total organic carbon in the loess-paleosol profile

It has been well documented that the properties of soil organic matter were greatly influenced by climate (precipitation and temperature) and vegetation conditions (Wang et al., 2004). For example, soil organic carbon content of forest steppe under a warm and humid climate was higher than arid grassland under a cold climate (Li & Wang, 2017). It has also been reported that the distribution of total organic matter content (TOC) in loess-paleosol sequences was closely correlated with paleoclimate changes, and paleoclimate was the key controlling factor of soil organic matter content (Stevenson, 1994). Our results showed that in the Chunhua loess-paleosol profile, total organic matter content (TOC) was higher in paleosol layers than in loess layers. The results could reflect that strong summer monsoon and abundant precipitation occurred during the formation periods of paleosols, which could lead to high coverage of aboveground vegetation and strong biological pedogenesis, and therefore resulted in the increases in TOC content in soil. The content of TOC in S0-1 was the highest in all soil layers, which was the result of long-term application of organic fertilizer. According to the environmental temperature trend curve recorded by snail fossils in the Luochuan loess-paleosol profile (Luochuan county, Shaanxi province, China) (Liu, 1985), from the early Pleistocene to the late Pleistocene, the climate gradually changed from warm and humid to cold and dry, and the vegetation environment experienced the evolution process of forest steppe-grassland-arid grassland. However, consistent with earlier results about the distribution of organic matter content in Luochuan loess-paleosol profile (Liu, 1985), our results showed that organic carbon content decreased with the increase of soil depth and tended to be stable in soil layers below the paleosol layer S8. This might be because during the long-term burial of the loess-paleosol sequence, the organic matter in loess and paleosol were continuously decomposed without the addition of extraneous organic carbon, which also led to the transformation from easily oxidized organic carbon to stable organic carbon and therefore resulted in the changes in composition of organic carbon fractions.

### Distribution characteristics of MOC/TOC in the loess-paleosol profile

It is well known that MOC refers to the organic carbon that combines with <53 µm particles, especially clay and silt with large surface area (Six, Elliott & Paustian, 2000; Feng et al., 2014), and MOC/TOC ratios reflect the relative amount of stable organic carbon in soil (Six et al., 2002). In our study, MOC/TOC ratios in all layers of the Chunhua profile were quite high. That is because during a long-term burial, soil active organic carbon, such as particulate organic carbon, is easily decomposed and therefore gradually decreased, while mineral-associated organic carbon is stabilized by fine soil particles (i.e., silt and clay) with a slow turnover rate. Therefore, the proportion of MOC kept at a relatively high level, which implies the importance of MOC in the loess-paleosol profile in exploring SOC dynamics on the Loess Plateau in the Quaternary. In addition, MOC/TOC ratios were higher in paleosol layers than in the underlying loess layers, which should be because the decomposition rate of POC increased under the warm and humid climate in paleosol formation periods, leading to the increase of MOC/TOC ratios. Our results also showed that MOC/TOC greatly differed in two sampling sites of loessial soil, and was significantly lower in S0-1 than in S0-2. This could mainly be attributed to human interference. S0-1 belongs to the tillage soil, in which the microaggregates produced by cultivation and fertilization could exert the accumulation and protection effect on soil particulate organic carbon, lowering the decomposition and mitigation rate of labile organic carbon. In general, the distribution of different soil organic carbon fractions significantly differed in the loess-paleosol profile, and MOC/TOC ratios may have great significance in indicating paleoenviromental and paleoclimatic evolution on the Loess Plateau in the Quaternary.

### Distribution characteristics of soil grain size and CaCO_3_ content in the loess-paleosol profile

In earlier studies, soil grain size characteristics and CaCO_3_ content have been widely recognized as paleoclimatic proxies and used for studying paleoenvironmental changes on the Loess Plateau. It has been documented that changes in clay content could reveal the intensity of the winter monsoon and indicate the strength of pedogenesis during loess deposition (Huang, Pang & Zhao, 2000). The clay contents in paleosol layers were higher than those in loess layers, which might be because during the formation period of the paleosol, the climate was warm and humid under the effects of the relatively weak winter monsoon and strong summer monsoon. Therefore, the eolian sediments were dominated by fine particulate matter, and the weathering intensity increased, which were conductive to the enrichment of clay. However, in the formation periods of the loess, the winter monsoon prevailed, leading to a cold and dry climate. Under such climate conditions, eolian sediments were dominated by coarse particulate matter, and the weathering and pedogenesis intensities were weakened, which decreased the clay content in the loess (Han, 1982). From Wucheng loess to Holocene loess in the Chunhua loess-paleosol profile, soil clay content gradually decreased, thus it can be inferred that from the early Pleistocene to Holocene, climate gradually changed from humid and warm to dry and cold, which is consistent with earlier results found in Luochuan profile, Shaanxi province (Liu, 1985).

Similar to soil grain size, CaCO_3_ content in soil is also closely related to climate change. CaCO_3_ content in soil was mainly influenced by the leaching intensity in the eolian sedimentation area, and the leaching degree mainly depended on local climate conditions, especially precipitation. Low rainfall amount could limit the dissolution and migration of CaCO_3_ in soil, while high rainfall amount promoted the dissolution and migration of CaCO_3_, which decreased the content of CaCO_3_ maintained in soil. In this study, the content of CaCO_3_ was higher in loess layers than in paleosol layers, which could be attributed to the cold and dry climate during loess formation periods. Under the conditions of low rainfall and cold temperature, the leaching and deposition of CaCO_3_ were not strong, with relatively high contents of CaCO_3_ left in loess layers. The lower content of CaCO_3_ in paleosol layers reflected good moisture conditions during the paleosol formation periods, for the abundant rainfall and warm temperature result in high leaching intensity of CaCO_3_. The CaCO_3_ content in S0-1 significantly differed with that in S0-2, which was mainly because of the artificial farming disturbance on S0-1. The long-term irrigation could lead to a greater leaching loss of CaCO_3_.

### The paleoclimatic significance of MOC/TOC in the loess-paleosol sequence

The results of this study supported our hypothesis, which demonstrated that compared to total organic carbon, MOC/TOC ratios showed more significant correlations with paleoclimatic proxies, such as soil particle size and CaCO_3_ content, in the Chunhua loess-paleosol profile. This implies the significance of MOC/TOC ratio in the loess-paleosol sequence for the indication of paleoclimate evolution. It can be inferred that during the formation periods of paleosols, the climate was warm and humid, the vegetation coverage was relatively high and water conservation capacity was improved, which promoted the leaching and migration of CaCO_3_ in soil and resulted in the decrease of soil CaCO_3_ content. Meanwhile, the weathering and pedogenesis intensities increased, and fine particles in soil, such as clay and silt, also increased, leading to the increases in MOC/TOC ratios. Consistent with our results, earlier studies showed that MOC/TOC ratio was positively correlated with the amount of fine fractions in soil, which could be greatly affected by soil texture and related climate (Dobarco & Miegroet, 2014; Cai et al., 2016). In contrast, under a cold and dry climate in the formation periods of loess, the sparse vegetation and weak water conservation capacity decreased the leaching and migration of CaCO_3_ and resulted in high amount of CaCO_3_ maintained in loess. At the same time, the intensity of pedogenesis was weak. Therefore, clay content in soil and Md (*ϕ*) was lower, in that way the amount of <53 µm particles decreased, which reduced the formation of MOC and decreased MOC/TOC ratios. Our results showed that MOC/TOC ratios in paleosol layers were higher than those in the underlying loess layers. It should be noticed that in the soil layers below the S8 paleosols layer of the Chunhua profile, MOC/TOC was more obviously correlated with paleoclimatic proxies, but represented different distribution characteristics with total organic carbon, which reflected that MOC/TOC was more suitable for indicating paleoclimate than total organic carbon, especially for the soil layers experiencing longer burial time.

Earlier results have shown that under various vegetation types, not only soil total organic carbon content, but also characteristics of soil organic carbon fractions, could be greatly different. For example, POC/MOC ratios showed significant differences under different vegetation types, which listed as follows: bare land¡shrub land¡grassland (Liao & Long, 2011). The results reflected that with vegetation succession, the moisture and temperature conditions of soil were gradually improved, and therefore POC increased more quickly in soil than MOC, leading to the increases of POC/MOC. However, different from the modern soil, the loess-paleosol profile was buried for a long time, and particulate organic carbon in soil was continuously decomposed due to its poor stability and high turnover rate. Finally, the contents of POC maintained in all loess and paleosol layers were almost the same, while MOC was limited by the surface of soil mineral, thus its losses were relatively low during long-term burial. Therefore, in the formation periods of paleosols, better moisture and temperature conditions of soil led to lower POC/MOC ratios, whereas in the loess formation period, poor moisture and temperature conditions resulted in lower proportions of MOC. Consistent with previous studies, our results also provided evidence that MOC/TOC ratios might be closely correlated with the conditions of climate and vegetation. In conclusion, the results of our study showed that the content and fractions of soil organic carbon had significant differences among different layers of the Chunhua loess-paleosol profile. MOC/TOC ratios showed significant correlations with paleoclimatic proxies, such as soil clay content, Md (*ϕ*) and CaCO_3_ content, especially in the soil layers below the S8 paleosol layer. Therefore, compared to total organic carbon, MOC/TOC ratios in different layers of the loess-paleosol profile can more directly reflect the responses of dynamics and stock of soil organic carbon to climate evolution, which have a great significance in studying paleoenvironment and paleoclimate changes on the Loess Plateau in the Quaternary.

## Conclusions

In conclusion, our results showed that total organic carbon content and mineral-associated organic carbon content, as well as MOC/TOC ratios, were higher in paleosol layers than in the underlying loess layers on the Chunhua loess-paleosol profile, which reflected the strong effects of the summer monsoon and the abundant precipitation during the formation periods of paleosols. However, different from total organic carbon, MOC/TOC had significant correlations with the paleoclimatic proxies, soil grain size (clay content, Md (*ϕ*)) and CaCO_3_ content in the Chunhua loess-paleosol profile, especially in soil layers below S8. Our study demonstrated that compared to total organic carbon, the distribution of MOC/TOC in loess-paleosol profile could more directly reflect the responses of stock and dynamics of soil organic carbon to climate evolution, and therefore could provide a better indication of paleoclimate evolution in the Quaternary, especially for the strata experiencing longer-term burial. Our results could not only provide new evidence from the aspect of soil organic carbon fraction composition for the reconstruction of paleoenvironment and paleoclimate on the Loess Plateau in the Quaternary, but also provide vital references for predicting the responses of the soil organic carbon pool to climate and vegetation changes in the future.

##  Supplemental Information

10.7717/peerj.4611/supp-1 Supplemental Information 1 Raw data

10.7717/peerj.4611/supp-2 Supplemental Information 2 The Chunhua loess-paleosol profile Phote credit: Qingqing Zhang.

## Additional Information and Declarations

### Data Availability

The following information was supplied regarding data availability: The raw data is provided in a Supplemental File.
