# Supplementary figures and images for: The distribution of organic carbon fractions in a typical loess-paleosol profile and its paleoenvironmental significance
Source: PeerJ. 2018 Apr 13;6:e4611. doi: 10.7717/peerj.4611 (PMC5900933; doi:10.7717/peerj.4611)

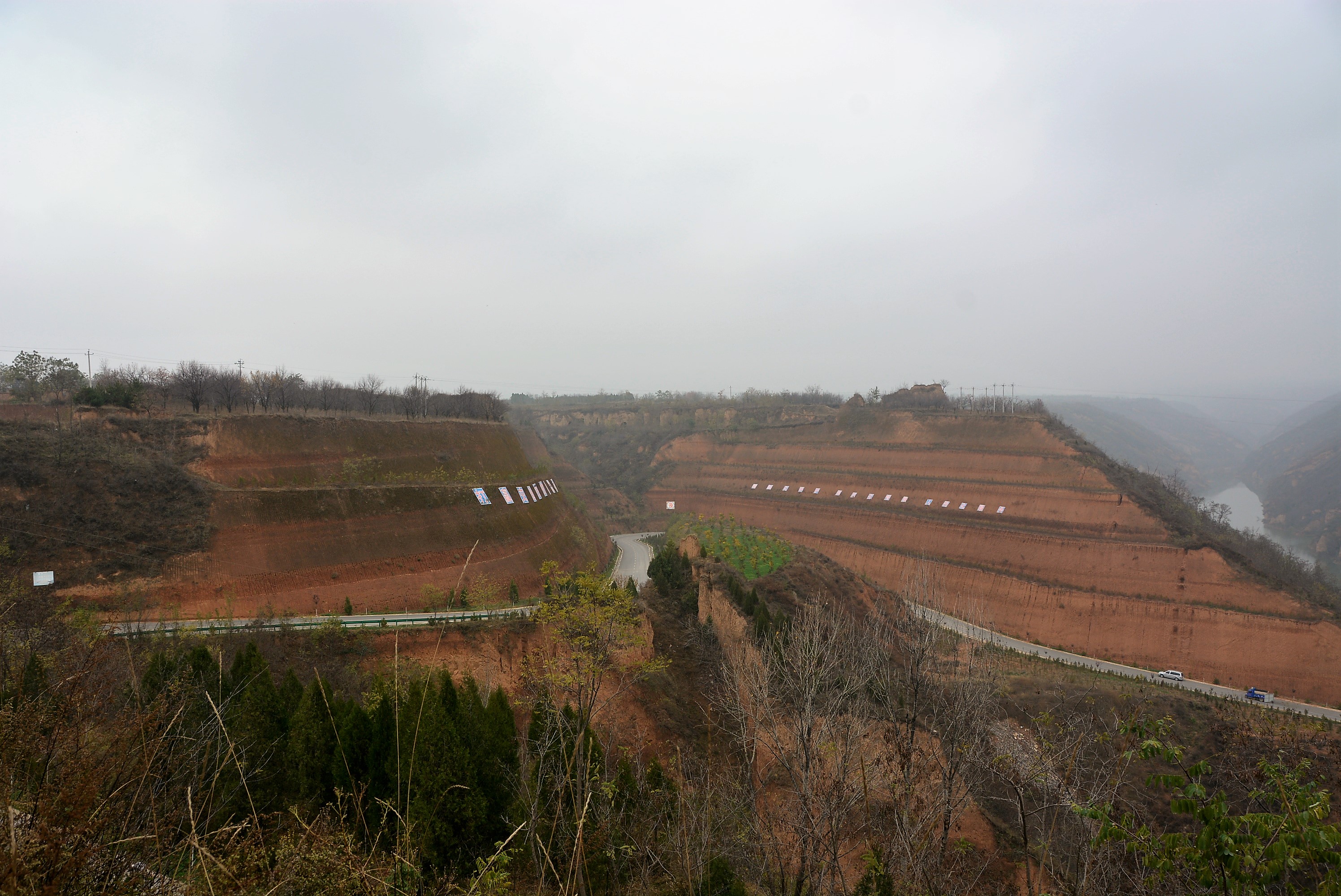

Supplement: Supplemental Information 2 — Phote credit: Qingqing Zhang. [file peerj-06-4611-s002.jpg]
